# Need vs. Financing Capability: Human Papillomavirus Vaccinations among Adolescents

**DOI:** 10.31557/APJCP.2019.20.10.2959

**Published:** 2019

**Authors:** Wiwin Lismidiati, Ova Emilia, Widyawati Widyawati

**Affiliations:** 1 *Department of Pediatric and Maternity Nursing,*; 2 *Department of Medical Education, Faculty of Medicine, Public Health and Nursing, Universitas Gadjah Mada, Indonesia. *

**Keywords:** HPV, cervical cancer, HPV vaccine, knowledge, financing

## Abstract

**Background:**

The incidence of Human Papillomavirus (HPV) infection and cervical cancer in adulthood may be prevented by HPV vaccination in adolescence. Currently, the HPV vaccination coverage rate in developing countries is about 15%. The reason for this low vaccination coverage is most likely due to a lack of information among adolescents and adults.

**Purpose:**

To explore adolescents, parents and teachers’ needs, obstacles, and expectations around the HPV vaccination.

**Methods:**

This research used a qualitative method with a focus group discussion. The research participants were divided into three groups: 21 female students, 17 parents, and 20 teachers. This research was conducted in junior high schools that have programs run by their adolescent reproductive health counseling information centers. The data were analyzed by employing content analysis.

**Results:**

HPV vaccination has not been made a priority for adolescents because: 1) There is a lack of available education about HPV and HPV vaccinations for adolescents, parents, and teachers. 2) The high cost for parents to vaccinate their children. 3) Adolescents, parents and teachers believe that the HPV vaccine needs to be administered to adolescents, but they feel that the vaccine is not affordable.

**Conclusion:**

It is important to consider a program which will provide accurate information about the HPV vaccination to the community, especially adolescents. Financial management, such as insurance or vaccination savings schemes, may be one way to overcome the problem of the HPV vaccination’s cost.

## Introduction

The World Health Organization (WHO) states that the Human Papillomavirus (HPV) prophylactic vaccine is highly effective when administered to 16-20 year-old females. The HPV vaccine is expected to prevent up to 70% of HPV incidence in vaccinated females (WHO, 2006). The HPV vaccination is most effective when administered to 9-13 year-old adolescents, and to 14-26 year-old non-vaccinated people before they have had sexual intercourse. Research shows that if administered in this way, the HPV vaccination may achieve a nearly 90% protection rate (Cunningham et al., 2015; Gallagher et al., 2018).

In 34 low- and low- to middle-income countries, the vaccination program is supported by the Global Alliance for Vaccines and Immunizations (GAVI). From 2013 to 2016, GAVI provided support to more than 20 countries eligible for a 2-year HPV vaccine demonstration project. In 2014, 1.2% of 10-14 year-old adolescents were expected to have had at least one dose of the vaccine administered (Bruni et al., 2014; Gallagher et al., 2018).

In Indonesia, the HPV vaccine is available and is recommended by the Indonesian Pediatric Society. However, at the national level, the administration of the HPV vaccination is still in its initiation process. The HPV vaccination is administered to female students in grades five (first dosage) and six (second dosage) at elementary schools through the School Children’s Immunization Month (BIAS) program (Arifah et al., 2017).

Low- to middle-income countries face some constraints in introducing the HPV vaccination to their people. These challenges include: historical/structural factors; programmed vaccinations; and delivery constraints (Gallagher et al., 2018). Other constraints found include socio-cultural, health system, political, and financial factors (Wigle et al., 2013). 

In Indonesia, constraints include the parents’ acceptance of the HPV vaccinations, the vaccine’s cost, the fear of side effects, the preferred location for HPV vaccination (Jaspers et al., 2011), the lack of access to service centers with adequate laboratory and health workers, and the requirement for repeated visits to the service center (Karneli et al., 2013). In addition, a lack of knowledge about the HPV vaccination, the vaccine’s safety, side effect considerations, and not being recommended by physicians or health workers are some of the other challenges found around the HPV vaccination’s administration (Arifah et al., 2017).

The research question is what are the needs, challenges and expectations of female students, parents and teachers for the HPV vaccination program?

## Materials and Methods

This qualitative research took place in junior high schools in Bantul and Sleman under the Adolescent Reproductive Health Counseling Information program in Yogyakarta Special Region.

*Study design and sample*

This focus group discussion was carried out from December 2017 to January 2018. The research design was qualitative using focus group discussion approach. This research sample consists of three groups: female junior high school students, parents, and teachers. The inclusion and exclusion criteria for the female students’ group included female students in grades seven and eight who were willing to be participants and who signed the informed consent form; the inclusion and exclusion criteria for the parents’ group included the parents of female junior high school students in grades seven and eight who were willing to be participants, and the inclusion and exclusion criteria of the teachers’group included female teachers who were actively teaching subjects during the research period and willing to be respondents.

The teachers’ group included teachers who teach school subjects and counseling teachers who have the important role of helping female students at school on a daily basis. Ten teachers were invited to the discussion from each school, and all of them were permitted to attend the discussions. Ten to 12 female students were invited from each school to the Focus Group Discussion (FGD); in all, 10 female students were present from Junior High School A and 11 female students were present from Junior High School B. The parents’ group was sampled using the criteria of those with female children in grades seven and eight at the same schools. 10 to 12 parents were invited from each school; six parents were present during the FGD activity from Junior High School A and 11 parents were present from Junior High School B.

*Data collection*

The FGD groups met in the same place as their respective schools. The FGDs were conducted in classrooms and laboratory rooms in the respective schools, and lasted for 60-90 minutes. Before starting the group discussions, the purpose of the FGD was explained to the participants and they were asked to provide their written consent through an informed consent form. The participants were told that any data would be kept confidential and that answer anonymity would be maintained. Each discussion group was led by one research facilitator, and an assistant was assigned to take notes and help manage the group. The group discussions were digitally recorded with the permission of the participants. 

The research instruments included prepared FGD instructions. The FGD instructions were arranged by the researcher pursuant to previous theories and related research studies. The FGD instructions contained some open-ended questions that were adapted to each discussion group. This research was approved by the Medical and Health Research Ethical Committee under number KE/FK/1100/EC/2017. 

**Table 1:** Characteristic of Adolescent’Participants

Adolescent Partisipant	Age	Characteristic
AP 1	13 years old	Eight grade junior high female students in Srandakan Bantul
AP 2	12 years old	Seventh grade junior high female students in Srandakan Bantul
AP 3	13 years old	Eight grade junior high female students in Srandakan Bantul
AP 4	12 years old	Seventh grade junior high female students in Srandakan Bantul
AP 5	13 years old	Eight grade junior high female students in Srandakan Bantul
AP 6	13 years old	Eight grade junior high female students in Srandakan Bantul
AP 7	14 years old	Eight grade junior high female students in Srandakan Bantul
AP 8	12 years old	Eight grade junior high female students in Srandakan Bantul
AP 9	12 years old	Seventh grade junior high female students in Srandakan Bantul
AP 10	13 years old	Eight grade junior high female students in Srandakan Bantul
AP 11	14 years old	Eight grade junior high female students in Ngaglik Sleman
AP 12	13 years old	Eight grade junior high female students in Ngaglik Sleman
AP 13	14 years old	Eight grade junior high female students in Ngaglik Sleman
AP 14	14 years old	Eight grade junior high female students in Ngaglik Sleman
AP 15	13 years old	Eight grade junior high female students in Ngaglik Sleman
AP 16	13 years old	Eight grade junior high female students in Ngaglik Sleman
AP 17	13 years old	Eight grade junior high female students in Ngaglik Sleman
AP 18	14 years old	Eight grade junior high female students in Ngaglik Sleman
AP 19	14 years old	Eight grade junior high female students in Ngaglik Sleman
AP 20	13 years old	Eight grade junior high female students in Ngaglik Sleman
PS 21	13 years old	Eight grade junior high female students in Ngaglik Sleman

**Table 2:** Characteristic of Parents’ Participants

Parents Participant	Age	Keterangan
PP1	42 years	Mothers of seventh grade junior high female students in Srandakan Bantul. She is an employed. Educational background was senior high school.
PP 2	37 years	Mothers of eight grade junior high female students in Srandakan Bantul. She is self employed. Educational background was senior high school.
PP 3	37 years	Mothers of seventh grade junior high female students in Srandakan Bantul. She is an employed. Educational background was junior high school.
PP 4	45 years	Mothers of eight grade junior high female students in Srandakan Bantul. She is self employed. Educational background was bachelor.
PP 5	41 years	Mothers of seventh grade junior high female students in Srandakan Bantul. She is in private sector. Educational background was senior high school.
PP 6	47 years	Mothers of eight grade junior high female students in Srandakan Bantul. She is self employed. Educational background was senior high school.
PP 7	38 years	Mothers of eight grade junior high female students in Ngaglik Sleman. She is in private sector. Educational background was senior high school.
PP 8	45 years	Mothers of eight grade junior high female students in Srandakan Bantul. She is an employed. Educational background was senior high school.
PP 9	39 years	Mothers of eight grade junior high female students in Ngaglik Sleman. She is an employed. Educational background was senior high school.
PP 10	40 years	Mothers of eight grade junior high female students in Ngaglik Sleman. She is an employed. . Educational background was senior high school.
PP 11	38 years	Mothers of eight grade junior high female students in Ngaglik Sleman. She is self employed. Educational background was Diploma.
PP 12	37 years	Mothers of eight grade junior high female students in Ngaglik Sleman. She is an employed. Educational background was Diploma.
PP 13	46 years	Mothers of eight grade junior high female students in Ngaglik Sleman. She is self employed. Educational background was senior high school.
PP 14	39 years	Mothers of eight grade junior high female students in Ngaglik Sleman. She is an employed. Educational background was vocational school.
PP 15	39 years	Mothers of eight grade junior high female students in Ngaglik Sleman. She is an employed. Educational background was vocational school.
PP 16	44 years	Mothers of eight grade junior high female students in Ngaglik Sleman. She is in private sector. Educational background was vocational school
PP 17	40 years	Mothers of eight grade junior high female students in Ngaglik Sleman. She is an employed. Educational background was Diploma.

**Table 3:** Characteristic of Teachers‘Participant

Teacher Participants	Usia	Keterangan
TP 1	43 years	Math at junior high school teacher in Srandakan Bantul
TP 2	44 years	Civilization Education at junior high school teacher in Srandakan, Bantul
TP 3	42 years	Biology at junior high school teacher in Srandakan, Bantul
TP 4	47 years	Math at junior high school teacher in Srandakan Bantul
TP 5	45 years	Social science at junior high school teacher in Srandakan Bantul
TP 6	46 years	Guidance counselor at junior high school teachers in Srandakan Bantul
TP 7	46 years	Social science at junior high school teacher in Srandakan Bantul
TP 8	43 years	Civilization Education at junior high school teacher in Srandakan, Bantul
TP 9	46 years	Math at junior high school teacher in Srandakan Bantul
TP 10	46 years	Guidance counselor at junior high school teachers in Srandakan Bantul
TP 11	44 years	Math at junior high school teacher in Ngaglik Sleman
TP 12	45 years	Gym at junior high scholl teacher in Ngaglik Sleman
TP 13	46 years	Civilization Education at junior high school teacher in Ngaglik Sleman
TP 14	43 years	Physics at junior high school teacher in Ngaglik Sleman
TP 15	44 years	Biology at junior high school teacher in Ngaglik Sleman
TP 16	43 years	Physics at junior high scholl teacher in Ngaglik Sleman
TP 17	44 years	Gym at junior high scholl teacher in Ngaglik Sleman
TP 18	45 years	Biology at junior high school teacher in Ngaglik Sleman
TP 19	44 years	Social science at junior high school teacher in Ngaglik Sleman
TP 20	46 years	Physics at junior high school teacher in Ngaglik Sleman

*Data analysis*

The recording of each discussion was transcribed by the researcher. The written notes made by the research assistant were added to the record. The research data were analyzed manually with the following qualitative research measures: 1) Transcribing the data collected from the FGDs and making notes during the data’s collection about data related to the informants. 2) Reconfirming the accuracy of the data with the participants and triangulating the data with the community health center officer in charge of the local junior high schools health units. 3) Reading all the data or transcripts for general ideas presented by the informants and any other necessary information. 4) Starting coding. 5) Using the coding process’s results to develop the theme for further analysis. 6) Presenting the description and theme in a qualitative narration. 7) Interpreting the data.

The themes were analyzed for each question during the FGD sessions with the three participant groups. 

## Results

There were 58 participants of the FGDs: 21 female students, 17 parents, and 20 teachers. The participants’ characteristics can be seen in Table 1. 

There were five themes namely: (1) Lack of understanding about human papillomavirus vaccine; (2) Difficult to explain to children and make embarrassment are the constrain arise in the health education; (3) Parents’limited funds for children’s human papilloma virus vaccination; (4) Insurance covering, discount and savings is the effort that parent’s thought to solve financial problems; (5) The constrain in female vaccine are expensive, low economy, not priority in household budget.

*Theme 1: Lack of understanding about human papillomavirus and the human papillomavirus vaccine*

Fifty of participants expected that adolescents would receive the HPV vaccinations, considering the future effects of exposure to HPV infection. However, most of the participants lacked knowledge about HPV, cervical cancer, and the HPV vaccine. Some participants identified the importance of health education and health promotion with regard to HPV and the HPV vaccine. They stated that they wanted adequate information about HPV, cervical cancer, and the HPV vaccine.

Parent Participant (PP12):* “I agree on socialization, but parents must be involved.”*

Adolescent Participant (AP7):* “Socialization at school to provide information of reproductive health and teenager relationship”*

Teacher Participant (TP1):* “Maybe the knowledge should be prioritized, since it must be a process, not something instant. Children’s source of funding is from their parents. When the parents have no understanding or knowledge of its benefits, it will be hard.”*

Teacher Participant (TP13):* It (socialization) is also needed, but through the biology subject, during the study of reproduction.”*

*Theme 2: Difficult to explain to children and make embarrassment are the constrain arise in the health education*

Parent Participant (PP9):* “How to communicate, how to tal*k about it? Will they understand?” 

Parent Participant (PP10):* “No courage to explain it, since the children cannot understand what we tell them.”*

Teacher Participant (TP11):* “Anything related to female matters is an embarrassment, even if it is a free check-up. The children will also get embarrassed.”*

Teacher Participant (TP12):* “Lack of awareness and thinking that everything is alright.”*

*Theme 3: Parents’ limited funds for children’s human papillomavirus vaccination*

Almost all the participants of the three groups disclosed constraints to buying the human papillomavirus vaccine. Most of them stated that even if many people wanted to buy the HPV vaccine for their daughters, they were unable to buy it because it is expensive. The parents’ group disclosed that the vaccine could be included in health insurance plans like the Social Insurance Administration Body (BPJS), which is an important factor in increasing the HPV vaccine’s acceptance. Some parent participants stated that the vaccine should not be expensive or there should be a discount for the HPV vaccine’s purchase. 

*Theme 4: Insurance covering, discount and saving is the effort that parent needed to solve financial problems were identified, as follows:*

Parent Participant (PP18):* “Is it covered by the government’s BPJS? Or is it covered by Jamkesda (Regional Health Insurance) or Jamkesmas (Social Health Insurance)?”*

Parent Participant (PP12):* “There is normally a discount, for example, buy two get one free.”*

Adolescent Participant (AP11):*”Yes, may be after saving for it.”*

Teacher Participant (TP9): *“Create a program at school which may be budgeted next year.”*

*Theme 5: The constrain in female adolescent vaccine are expensive, low economy, not priority in house hold budget*

Parent Participant (PP1/PP6):* “The objection is about the high cost. I think it is too much for us in the village. We are in the village, and thus it is too expensive”*

Teacher Participant (TP10) :*“Parents are of a low economic status, from the perspective of their education and employment, 50% have a weak economy.”*

*A*dolescent Participant (AP7):* “There are still many necessities for school tasks.”*

## Discussion

Adolescents, parents, and teachers have limited knowledge about HPV, cervical cancer, and the HPV vaccine; thus, the need for health education and the socialization of HPV, cervical cancer, and the HPV vaccine is an important factor in changing behavior toward the HPV vaccination. Not much progress has been made by Indonesian health workers with the socialization of HPV, and getting people to have the HPV vaccine to prevent cervical cancer. This is confirmed by the results of an interview with a community health center worker, who stated that the HPV vaccine is not a priority program and that it is not the community health center’s policy to socialize it in the adolescent reproductive health concern (PKPR) program. Currently, the PKPR program at community health centers only socializes the issues of older marital age, an early introduction to sex, and adolescent anemia. The lack of recommendations by health workers when socializing the HPV vaccine was also found by Cartmell et al., 2018, which stated that factors contributing to the lack of information provided about the HPV vaccine include: 1) The lack of awareness among some pediatricians and general practitioners about the HPV vaccine directive. 2) Service providers are uncomfortable discussing the topic. Other researchers state that the reason for the lack of parents’ knowledge about HPV and the HPV vaccination, as well as for the parents’ concern about the side effects, is that there is no recommendation for it by health workers (Morales-Campos et al., 2013; Fernández et al., 2014; Masika et al., 2015). This is confirmed by previous research studies (Reiter et al., 2011; Bartolini et al.,2012) proposing that health workers and teachers are credible sources of information who could provide significant support for an HPV vaccination campaign. Recommendations from physicians is the consistent key predictor of the HPV vaccine’s acceptance (Rosenthal et al., 2011).

In Indonesia, parents have a low level of knowledge about HPV, the HPV vaccination, and cervical cancer topics in general (Jaspers et al., 2011). Only about 16.6% of parents have heard about HPV; about 15.8% have heard of the HPV vaccine; and more than 40% lack understanding about HPV, the HPV vaccination, and cervical cancer. This percentage is nearly equal to that found in Malaysia and Singapore. There, 12.2% and 20.0% (respectively) have heard about HPV, and 10.5% and 15.8% (respectively) have heard about the HPV vaccine (Jaspers et al., 2011; Sam et al., 2009). According to these research results, we can conclude that greater information is needed about HPV, cervical cancer, and HPV vaccine topics, considering that the knowledge levels are still low.

This is also confirmed by research (Dempsey and Zimet, 2015; Degarege et al., 2018) stating that health education programs should target the parents and extended family members, so as to increase the coverage rate for future vaccination programs. Parental and school involvement strategies are also an important factor for improving the school vaccination program for adolescents (Whelan et al., 2014).

In this research, both the parents’ and teachers’ groups stated that they face constraints in providing their daughters with education about sexual matters. The cultural/taboo factor prevents them from providing adolescents with sexual information. Reproductive and sexual health education is a sensitive topic, which needs advocacy to allow it to be provided to young people and the general public. So far, sexual and reproductive health education in schools is incomprehensive and not relevant to the actual sexual behavior and risks adolescents face. This implies the students have limited knowledge about reproductive health in general. Sexual and reproductive health education provided in schools tends to see adolescent reproductive and sexual health issues as merely biological phenomena, and often considers adolescent sexuality as something taboo and dangerous, which should be controlled through moral and religious discourse instead of education (Pakasi and Kartikawati, 2013). The results of the research conducted by Francis et al., (2011) also state that cultural norms and gender form the clearest response with regard to sexual health issues in communications about sexuality between mothers and children.

The results of this research also identify constraints in financing for the HPV vaccination. The high price of the vaccination, combined with no recommendation for it from physicians, is the most influential factor in the HPV vaccination’s acceptance. Most of the respondents are from the middle to lower socio-economic groups. Reproductive health savings for the HPV vaccinations’ financing is an alternative method for increasing the HPV vaccination’s coverage in schools. The research results also show that the three groups of female students, parents, and teachers expect the HPV vaccination program to be free. If the program was free, the HPV vaccination coverage rate would be higher. The triangulation result with health workers states that the community health centers’ current program for cervical cancer prevention is still in its early stages, with Visual Inspection with Acetic Acid (VIA) examinations, and there is no socialization of the HPV vaccine as yet. The reason for this is that the program and activity have not yet been included in the regional regulations. 

The Minister of Health (2016) stated that the HPV vaccine has just been initiated as part of the government’s national program, but it is not a national program for vaccination, so the HPV vaccination’s cost is still high. The government’s currently prioritized measure is to mobilize resources to strengthen the health system and buy the HPV vaccine. Indonesia has not taken innovative measures, in terms of financing, for introducing the HPV vaccination. Meanwhile, Malaysia has included the HPV vaccine as a priority in its adolescent program, through its school health program (Ezat and Syed, 2011). 

One main constraint causing parents not to accept the administration of the HPV vaccine in Indonesia is the cost. The HPV vaccination’s cost is not affordable for most Indonesians. The people believe that the government should either fund the vaccination or there should be joint funding between the government and parents for the HPV vaccinations (Arifah et al., 2017; Jaspers et al., 2011; Karneli et al., 2013).

Education about HPV, cervical cancer, and the HPV vaccine is recommended as necessary for adolescents, parents, and teachers. The results of research into the parent and teacher groups states that HPV, cervical cancer and HPV vaccine education should also be provided by involving parents. Parental involvement, through meetings at school, is required since parents make the decisions about their children’s vaccinations. Parental involvement in school meetings is an important strategy to educate about HPV, cervical cancer, and the HPV vaccination (Remes et al., 2012). It is important to focus on improving the overall level of knowledge. Similarly, consideration should be made for an alternative to the HPV vaccination’s financing, such as an adolescent reproductive health savings scheme, in order to maximize the HPV vaccination’s coverage rate.
